# In-situ datasets of important physical and bio-chemical parameters in the continental shelf of the northern Bay of Bengal

**DOI:** 10.1016/j.dib.2021.106947

**Published:** 2021-03-15

**Authors:** Most Israt Jahan Mili, Md Kawser Ahmed, Md Masud-Ul-Alam, Md Hasnain, Md. Ashif Imam Khan, Rupak Loodh, Abdullah-Al Hasan, Kazi Belayet Hossain, Sultan Al Nahian

**Affiliations:** a Department of Oceanography and Hydrography, Bangabandhu Sheikh Mujibur Rahman Maritime University, Pallabi, Mirpur-12, Dhaka 1216, Bangladesh; b Department of Oceanography, University of Dhaka, Dhaka-1000, Bangladesh; c Lieutenant commander, Bangladesh Navy, Dhaka, Bangladesh; d Bangladesh Oceanographic Research Institute, Ramu, Cox's bazar, Bangladesh; e Department of Marine Fisheries and Oceanography, Patuakhali Science and Technology University, Patuakhali, Bangladesh; f State Key Laboratory for Marine Environmental Science, Xiamen University, China

**Keywords:** CTD, Physical parameters, Heavy metals, Total organic carbon, Elements (CHNS), Nutrients, chlorophyll, Bay of Bengal

## Abstract

Data equipped with this article were collected from Northern Bay of Bengal (NBoB) wrapping both the eastern and western coast for CTD and sediment samples and only the eastern coast for water sampling. In-situ data of physical parameters, heavy metals, elements, Total Organic Carbon (TOC), nutrients, chlorophyll-a and phaeopigment were sampled across the shallow continental shelf. These data were assembled from 15 CTD points, 76 water samples, and 10 surface sediment samples adjacent to Bangladesh coast. Vertical CTD profiles were collected for Temperature ( °C), Salinity (PSU), Density (kg *m*^−3^), Turbidity (NTU), Fluorescence (mg *m*^−3^), and Dissolved Oxygen (DO, mg/l). Heavy metals (mg/l) of water column enlisted as Calcium (Ca), Cadmium (Cd), Copper (Cu), Cobalt (Co), Iron (Fe), Manganese (Mn), Magnesium (Mg), Nickel (Ni), Lead (Pb), and Zinc (Zn). Total Organic Carbon (TOC) was measured as Non-Purgeable Organic Carbon (NPOC) in ppm. Measurements of Chlorophyll - a, Nitrate, Nitrite, Phosphate, Ammonia, Silica and Phaeopigment were taken from 76 water sampling points. The survey was conducted with the assistance of a fishing vessel ‘Agro food-4 ‘of ‘Sea Resource Ltd.’ lengthening a fishing period from January to February (in winter), 2016. SBE 19 plus V2 CTD machine was deployed for sampling of vertical physical features, Niskin sampler of HYDRO-BIOS consisting of a non-metallic interior was used to collect water sample. Sediment was collected by Van Veen Grab sampler with built-in messenger. Water samples were analyzed following the standard procedure in the laboratory to access in-situ data. The shallow coastal and offshore regions of Bangladesh support for vast biological resources to its adjacent inhabitants. Therefore, understanding the influence of physico-chemical properties on other biological resources in coastal ecosystem is a crucial one to investigate. However, the shelf region of the BoB has a lack of in-situ baseline or reference data to compare with in terms of ocean biogeochemistry. Thus, these datasets can be utilized for further reference and also in validating other remotely-sensed physico-chemical parameters in this region.

## Specifications Table

**Table:** 

Subject	Earth and Planetary Sciences: Oceanography
Specific subject area	Physico-chemical oceanography, Metals, Elements, Seawater, Sediment, Biogeochemistry, Nutrient dynamics, Coastal process
Type of data	Tables, Figures, Graphs, Files
How data were acquired	In-situ data from CTD casting in the field for temperature, salinity, density, turbidity, fluorescence and DO. In-situ laboratory measurement of elements from sediment sample and metals and TOC in water sample. Laboratory measurement of Nitrate, Nitrite, Phosphate, Ammonia, Silica, Chlorophyll - a, and Phaeopigment. Instruments: i) CTD Machine, ii) Grab sampler iii) ‘Niskin’ water sampler, iv) CHNS element analyzer, v) TOC analyzer, vi) Flame AAS vii) Spectrophotometer. Model of the instruments used: i) Sea-Bird Electronics CTD Machine (SBE 19 plus V2), ii) Van Veen grab sampler, iii) 2.5 L model, HYDRO-BIOS Apparatebau GmbH, Altenholz – Germany, iv) VarioMicro V1.6.1, GmbH, Germany, v) TOC – VCPH, Shimadzu, Japan, vi) DR6000™ UV VIS, Hach Company, USA.
Data format	Raw, Analyzed, Filtered
Parameters for data collection	Station's positions (latitude and longitude), Tidal condition, Time of sampling, Depth, Temperature, Salinity, Density, Turbidity, Fluorescence, DO, Carbon, Hydrogen, Nitrogen, Sulfur, CH ratio, CN ratio, Total Organic Carbon (TOC), Calcium (Ca), Cadmium (Cd), Copper (Cu), Cobalt (Co), Iron (Fe), Manganese (Mn), Magnesium (Mg), Nickel (Ni), Lead (Pb), Zinc (Zn), Nitrate (NO_3_^−^-N), Nitrite (NO_2_^−^), Phosphate(PO_4_^3-^), Ammonia (NH_3_—N), Silica (SiO_2_), Chlorophyll - a, Phaeopigment.
Description of data collection	These in-situ data were obtained on-board during a routine fishing operation of ‘Sea Resource Ltd’, Bangladesh. Nutrients and chlorophyll data were collected at pre-determined 76 points, Physical parameter data were obtained on-board by CTD casting at 15 points, metals and TOC were analyzed from 30 water sample stations and CHNS from 10 sediment sample points. Sample of water and
	sediment were preserved on-board following standard procedures prior to analysis. Sampling period extended from 18 January to 11 February 2016, covering shelf area of northeastern part of the BoB.
Data source location	Region: Continental shelf of the northern BoB, Country: Bangladesh, Latitude, and longitude (GPS coordinates) for collected samples/data: (20.00 °N - 21.20 °N and 89.37 °E - 92.20 °E).
Data accessibility	With the article

## Value of the Data

• CTD data is key to investigate physical processes in shallower coastal BoB. Productivity can be perceived by in-situ chlorophyll-a, whereas, phaeopigment could be used as an indicator of phytoplankton bloom. Nutrients are inherent for productive zone detection and metals are prime to assess water quality as well as element cycling. In addition, major elements of sediment are crucial to identify the cycle path through the water column, atmosphere, and sediment.
• Overall information from the spatial distribution of the obtained nutrients and chlorophyll would result in decision making to the fishing community in the nearby fishing ground. Entirely, these distinct in-situ data can assist to perform ecosystem and biogeochemical modeling. Moreover, to validate remotely sensed data in this region.
• Physical parameters can be further related to detect mixed, barrier and isothermal layer thickness. Availability of limiting nutrients, such as nitrate, phosphate, and silica would implement in estimating phytoplankton growth, abundance, and biodiversity. Silica, would act as a tracer for the biogeochemical cycle, upwelling and carbon sequestration. Aquatic ecosystem's health can be indicated by the presence of heavy metals in the seawater.

## 1 Data Description

**Fig. 1:**
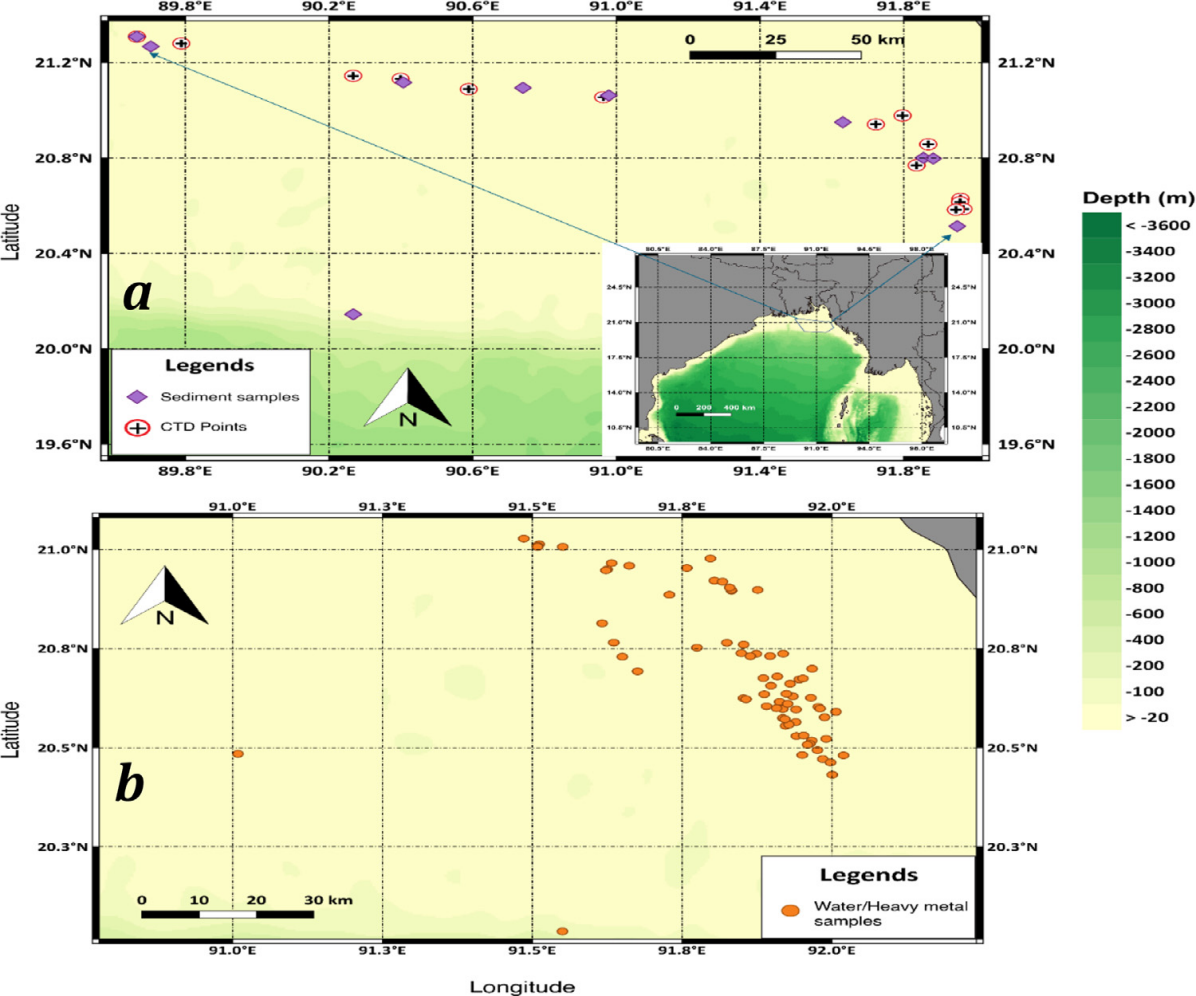
The Bay of Bengal, northern part of tropical Indian Ocean, sampling points covering shallower contental shelf area of both eastern and western coast of Bangladesh. In the above figure, (a), is for Sediment with purple diamond while, the “plus” signs represent points for CTD profiles and (b) are water samples illustrated with circles marked orange accordingly located near the northern Bay of Bengal. Bathymetry data were taken from Esri, GEBCO, NOAA, National Geographic, Garmin. Deep-green parts of the map represent a deeper sea while shallow seas are yellowish-green.

**Fig. 2:**
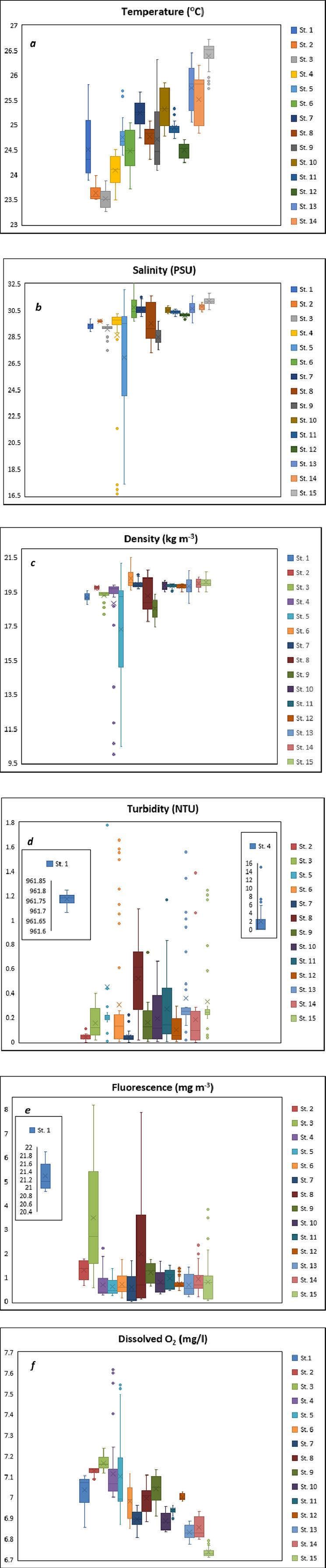
Boxplot of vertical physical parameters values from CTD measurements. Minimum, 1st quartile (Q1), median, 3rd quartile (Q3), and maximum value are distributed in the figure, ‘a’ - temperature, ‘b’ – salinity, ‘c’- density, ‘d’ – turbidity, ‘e’ – fluorescence, ‘f’ – dissolved O_2_. Turbidity has much higher value in case of station 1 and 4, therefore, incorporated separately into the boxplot of turbidity. Fluorescence has a much higher concentration in station 1, therefore, incorporated separately in boxplot of fluorescence.

One of the largest bays in the world, the Bay of Bengal (BoB), is located at the north-eastern wing of the Indian ocean. This bay ensembles higher magnitudes of changes in the aspect of thermodynamic parameters, compared to its neighboring Arabian Sea or the northern Indian Ocean [1]. Yet, a lack of collaborative research and research vessel, as well as paucity and unavailability of observational data [2], restricts through and continuous monitoring of these physico-chemical dynamics. The input of a large quantity of freshwater from major rivers [3] have a dominant influence on physico-chemical properties, therefore, needs to analyze with in-situ data of this region. Large fluctuations in physico-chemical and biological parameters have accelerated from land derived nutrients to the complex coastal ocean over the past decades [4]. Significant stress on coastal habitats has been exerted by multitude of physical, chemical, and biological processes [5], [6], [7], [8]. Partial CTD data of this manuscript was used in Hossain et al., 2018 [9]. Contributing to the pre-existing data gaps to study important physico-chemical processes, this article presents data of Temperature ( °C), Salinity (PSU), Density (kg *m*^−3^), Turbidity (NTU), Fluorescence (mg *m*^−3^), DO (mg/l), Carbon (%), Hydrogen (%), Nitrogen (%), Sulfur (%), C:N ratio, C:H ratio, Total Organic Carbon (ppm), Calcium (mg/l), Cadmium (mg/l), Copper (mg/l), Cobalt (mg/l), Iron (mg/l), Manganese (mg/l), Magnesium (mg/l), Nickel (mg/l), Lead (mg/l), and Zinc (mg/l) (Tables 1, 2, and 4 & Fig. 2). Placed adjacent to one of the largest deltaic systems in the world, with more than 1.7 billion tons of sediment flowing downstream yearly [10], makes the NBoB an interesting place for studying nutrients distribution. Recent studies propounded that the BoB might be more productive than previously estimated [11]. A better understanding of such discoveries is only possible by more in-situ datasets in the respective locations. Keeping that in mind, we present Nitrate (mg/L), Nitrite (mg/L), Phosphate (mg/L), Ammonia (mg/L), Silica (mg/L), Chlorophyll – a (mg/m^3^) and phaeopigment (mg/m^3^) in this article (Table 5 & Fig. 4). Though the study area covers the continental shelf region of the northern BoB, it is focused on the northeastern part of the BoB, which contains a total of 15, 76 and 10 points sequentially for CTD, water and sediment samples that spanned between 18 January 2016 to 11 February 2016 (Fig. 1). Sea-Bird CTD (SBE 19 plus V2) was maneuvered for taking profiles of physical parameters. Water samples, each with a volume of 500 ml was collected from each sampling point using the ‘Niskin’ Water Sampler, which is fully made of PVC and its interior is free from metal parts to avoid contamination with built-in messenger. Additionally, we deployed Van Veen grab sampler to extract sediment from the bottom of each station. Finally, concentrations of individual parameters from water and sediment samples were further analyzed at CARS (center for Advanced Research in Science) at the University of Dhaka using TOC analyzer (TOC – VCPH), Flame Atomic Absorption Spectrometry (Flame AAS) and CHNS element analyzer. Concentrations of individual nutrients were extracted from the obtained water sample and were further analyzed at oceanography lab at the University of Dhaka using DR6000™ UV VIS Spectrophotometer with built-in RFID Technology (Hach Company, USA). This model's scanning capability covers from UV to visible Spectrum, and includes 250 methods that were pre-programmed for water testing. Accordingly, these concentration data and physico-chemical parameter's values are represented in tabular format, comma-separated value (*.csv) and (*.txt) file.

**Table 1:** Outline of physical parameter's data from CTD cast, including date of sampling, coordinates, tidal context, ‘mean’ value, median and ‘standard deviation’ (SD) for each station.

		Coordinates			Temperature (°C)			Salinity (PSU)			Density (Kgm^−3^)			Turbidity (NTU)			Fluorescence (mgm^−3^)			DO (mg/l)		
St. no.	Date	Latitude (N)	Longitude (E)	Tide	Mean	Median	SD	Mean	Median	SD	Mean	Median	SD	Mean	Median	SD	Mean	Median	SD	Mean	Median	SD
**1**	20/01/16	20.950017	91.63075	Low	24.505	24.308	0.6	29.326	29.360	0.24	19.218	19.174	0.223	961.758	961.765	0.03	21.28	21.162	0.337	7.039	7.072	0.072
**2**	27/01/16	20.62895	91.9584	Low	23.640	23.568	0.157	29.709	29.701	0.072	19.752	19.759	0.077	0.042	0.042	0.028	1.324	1.406	0.407	7.129	7.137	0.017
**3**	27/01/16	20.614917	91.957133	Low	23.524	23.515	0.194	29.098	29.212	0.430	19.314	19.430	0.316	0.117	0.122	0.159	3.509	2.733	2.502	7.168	7.161	0.032
**4**	29/01/16	20.8575	91.8684	Low	24.089	24.112	0.314	28.666	29.799	3.490	18.834	19.697	2.415	1.774	0.203	3.327	0.704	0.407	0.548	7.117	7.066	0.152
**5**	29/01/16	20.585917	91.9664	Low	24.757	24.681	0.282	26.946	29.531	4.246	17.339	19.224	3.114	0.043	0.198	1.409	0.620	0.473	0.362	7.105	6.996	0.191
**6**	30/01/16	20.768967	91.835967	High	24.484	24.490	0.418	30.773	30.449	0.95	20.302	20.055	0.579	0.172	0.129	0.54	0.735	0.603	0.449	6.982	6.993	0.083
**7**	2/2/2016	20.582983	91.9455	Low	25.244	25.261	0.257	30.608	30.509	0.341	19.954	19.889	0.199	0.042	0.035	0.040	0.589	0.457	0.573	6.897	6.899	0.041
**8**	3/2/2016	20.977667	91.797	High	24.769	24.839	0.210	29.497	29.175	1.442	19.256	18.884	1.025	0.522	0.617	0.276	2.000	0.707	2.273	6.998	7.008	0.068
**9**	4/2/2016	21.054617	90.9639	Low	24.724	24.477	0.666	28.529	28.381	0.647	18.547	18.589	0.576	0.162	0.128	0.167	1.212	1.293	0.400	7.044	7.054	0.068
**10**	5/2/2016	21.3085	89.665333	High	25.325	25.341	0.387	30.546	30.504	0.208	19.886	19.972	0.236	0.192	0.114	0.199	0.835	0.744	0.429	6.890	6.883	0.042
**11**	5/2/2016	21.279933	89.789183	High	24.949	24.929	0.156	30.371	30.413	0.136	19.862	19.910	0.123	0.270	0.142	0.293	0.972	1.120	0.391	6.941	6.944	0.016
**12**	6/2/2016	21.131333	90.3995	Low	24.492	24.532	0.156	30.149	30.211	0.139	19.830	19.869	0.140	0.100	0.054	0.099	0.746	0.679	0.224	7.005	6.998	0.015
**13**	7/2/2016	21.144283	90.267517	Low	25.752	25.828	0.459	30.660	30.640	0.554	19.841	19.788	0.537	0.360	0.255	0.333	0.706	0.558	0.424	6.835	6.831	0.036
**14**	8/2/2016	21.08825	90.589333	High	25.522	25.824	0.480	30.781	30.833	0.216	20.002	19.954	0.266	0.184	0.097	0.292	0.945	0.702	0.589	6.858	6.832	0.051
**15**	11/2/2016	20.941117	91.72265	High	26.399	26.515	0.286	31.232	31.189	0.311	20.071	19.988	0.306	0.331	0.245	0.305	0.826	0.808	0.857	6.738	6.730	0.024

**Table 2:** Positions of sediment samples point including date, depth of each point, CHNS (Carbon, Hydrogen, Nitrogen, Sulfur) value of surface sediment, including CN ratio and CH ratio. All values are in percent (%) relative to dry weight.

			Coordinates							
St. no.	Date	Depth (m)	Latitude (N)	Longitude (E)	N (%)	C (%)	H (%)	S (%)	C/N ratio	C/H ratio
**1**	20/01/16	58.6	20.95001667	91.63075	2.42	0.65	0.397	0.125	0.2687	1.6364
**2**	28/01/16	46.4	20.80035	91.85511667	2.66	0.63	0.266	0.135	0.2369	2.3669
**3**	29/01/16	40.3	20.7976	91.88226667	2.81	0.62	0.142	0.107	0.2216	4.4029
**4**	1/2/2016	60	20.51378333	91.94916667	2.29	0.9	0.439	0.142	0.395	2.0589
**5**	4/2/2016	27.9	21.06183333	90.97916667	2.87	0.61	0.242	0.134	0.2129	2.5223
**6**	5/2/2016	46.5	21.26743333	89.7042	1.52	0.64	0.467	0.108	0.4227	1.3761
**7**	5/2/2016	41.3	21.30855	89.66521667	3.41	0.54	0.052	0.108	0.1573	10.2899
**8**	6/2/2016	42	21.11625	90.40726667	2.17	0.49	0.271	0.008	0.2235	1.7931
**9**	7/2/2016	42.5	20.14458333	90.26778333	1.98	0.55	0.278	0.091	0.2771	1.9707
**10**	8/2/2016	28.3	21.09386667	90.74021667	2.26	0.49	0.195	0.106	0.2176	2.5272

Relative error					6.830	6.074	15.031	11.347	10.036	27.203

## 2 Experimental Designs, Materials, and Methods

### 2.1 Data collection

In-situ data were gathered on-board, during a cruise that lasted for 25 days from 18 January to 11 February (categorized in winter) at the time of fishing operation of ‘Agro Food-4′ of ‘Sea Resource Ltd.’ having a dimension of a fishing-trawler 42:11:13 for length: wide: height accordingly. Data for physical properties were collected on-board by CTD measurement. Preserved sample for nutrients, chlorophyll, TOC, heavy metals, and elements were analyzed further in the laboratory after transportation. The depth of each station was determined by SIMRAD sonar. ‘Niskin’ water sampler having 2.5 L capacity of HYDRO-BIOS with built-in messenger was operated manually for water sample collection which had a non-metallic interior to avoid contamination with metals. Although the sampling depths varied with site conditions, most samples were attained at 1 m, 2 m, 3 m, 5 m, and some samples at 10 m, 15 m, 20 m, 25 m with the maximum depth at 35 m. Sediment samples were mostly varied from 40 m to 60 m contour lines. Polypropylene bottles were used for sample collection. However, it was cleaned earlier with 1:1 dilute hydrochloric acid, rinsed with Milli Q water and finally dried up. Collected water samples were labeled in bottles and kept in ice for preservation until those were transported to the laboratory for detailed biochemical analysis.

### 2.2 Lab procedures

#### 2.2.1 CTD data collection

Temperature was measured from 15 different sampling stations during the cruise from in-situ observations by a Sea-Bird Electronics CTD Machine (SBE 19 plus V2). SBE 19plus CTD can measure with an accuracy of temperature (± 0.005 °C). The resolution of temperature from this device is 0.0001. Temperature was measured in ( °C). SBE 19 plus V2 CTD has a resolution of conductivity 0.00005 (most oceanic water: resolves 0.4 ppm in salinity), 0.00001 (freshwater: resolves 0.1 ppm in salinity). Salinity was measured in PSU (Practical Salinity Unit). Salinity and Density (б-t, kg *m*^−3^) data were derived using SBE data processing software (SBE Data Processing Win-32). Fluorescence and turbidity were measured by WET Labs ECO-FLNTU sensors attached with the CTD machine. Fluorescence was measured in mg/m^3^ and turbidity was measured in NTU. For safety instruction, SBE 19 plus V2 CTD was submerged for priming of the pump below the sea surface for each profile before its sensor started reading. For this reason, in-situ measurement and parameters were obtained from 1 m depth instead of the sea surface. For all parameters priming data were removed using processing software, but the first scan count of priming was determined by another software Seasave V7. To communicate CTD with PC and for downloading data Seaterm V2 software was used. The unprocessed data was in a hex file (.hex) and the data was taken in ASCII format after processing. The mean value and standard deviation of each vertical physical parameter's profiles are shown in (Table 1) as a tabular form. The statistical distribution of data has been manifested in (Fig. 2).

#### 2.2.2 Element analysis (CHNS)

Sediment samples were dried first and the analysis was carried out with CHNS element analyzer at CARS (center for Advanced Research in Science), University of Dhaka, Bangladesh. Analyzer VarioMicro V1.6.1, GmbH, Germany model was used to perform the measurement. Assay specification for analysis was: Sample pan: Tin boat, Combustion temperature: 1150 °C, Reduction temperature: 850 °C, Gas flow rate: Helium – 200 mL/min, Oxygen −14 mL/min, Time for analysis: 800 s/sample, Detector: Thermal conductivity detector (TCD). All values are in percent relative to dry weight. Measured particulars have been conveyed in (Table 2).

#### 2.2.3 TOC measurement

**Fig. 3:**
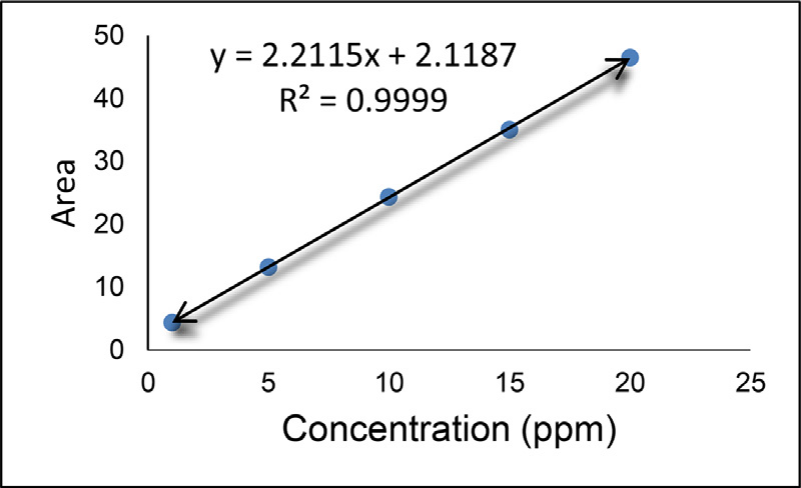
Concentration for standard curve taken as 0, 5, 10, 15, 20, 25 (ppm) for TOC reference value which has a significant ‘R’ of 0.9999.

Liquid samples were used for analysis. Assessments were carried out with TOC analyzer, Shimadzu, Japan (Model: TOC – VCPH). The system was TOC-regular sense, instrument option: TOC/ASI/IC unit, catalyst: regular sensitivity. Zero water required for blank preparation that refers to a standard solution with zero concentration of TC (Total Carbon) or IC (Inorganic Carbon). Zero water also refers to the water used to prepare standard solutions. Standard solution for TC: weighted 2.125 g of potassium hydrogen phthalate was dried at 105–120 °C for about 1 hour and then cooled in a desiccator. Therefore, it dissolved in zero water to leveled off up to 1 L, further, the required concentration of standard solutions was prepared with this stock solution. Standard solution for IC: previously dried (using silica gel desiccator) 3.5 g sodium hydrogen carbonate and 4.41 g sodium carbonate (dried for 1 hour at 280–290 °C and cooled in a desiccator) was dissolved in zero water (final volume 1 L) and required concentrations were prepared. Preparation of standard curve is shown in (Fig. 3).

**Fig. 4:**
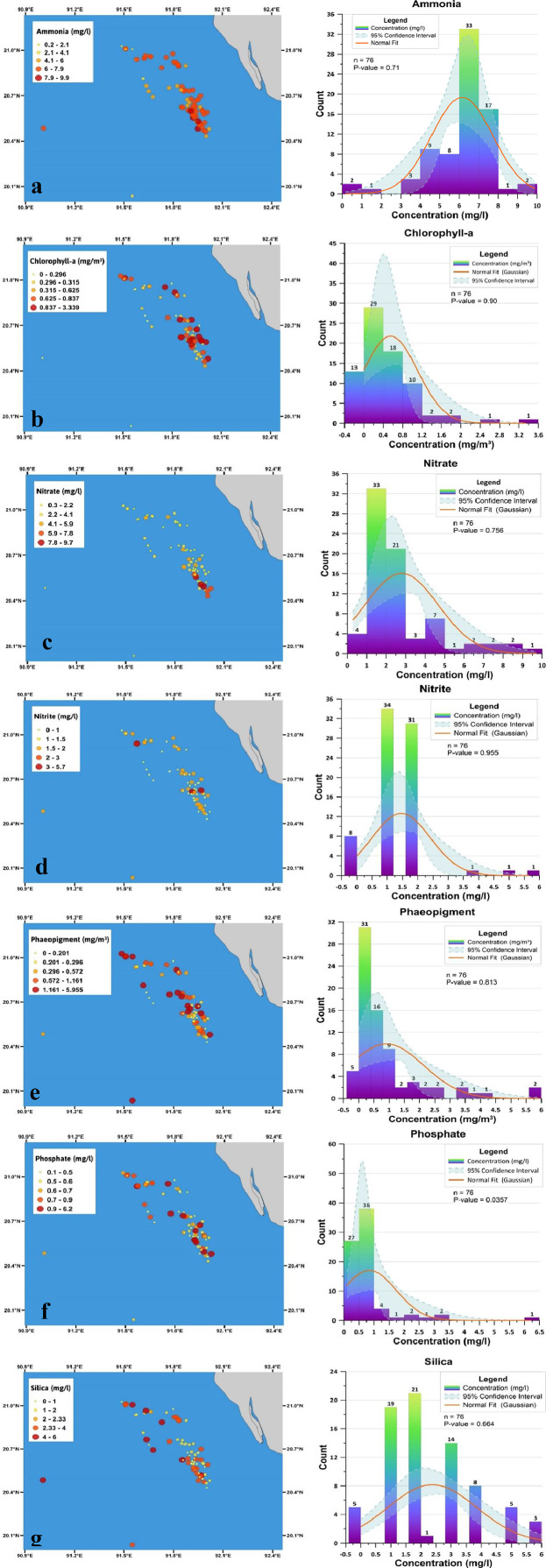
Spatial distribution of measured biochemical parameters (left) and the associated histograms (right) of (a) Ammonia (mg/L), (b) Chlorophyll (mg/m3), (c) Nitrate (mg/l), (d) Nitrate (mg/l), (e) Phaeopigment (mg/m3), (f) Phosphate (mg/L), (g) Silica (mg/l), where larger and deeper red circle illustrated higher concentration of nutrients while lighter yellow smaller circle indicates the opposite. Histograms were fitted to normal Gaussian distribution (orange line) with a 95% confidence interval (transparent light blue-green). Yellowish bars in histograms show higher frequency whereas the purple regions contain low frequency.

#### 2.2.4 Metal analysis

Preserved water samples were kept in room temperature until metal content analysis by Flame AAS (Atomic Absorption Spectroscopy). Calcium (wavelength 422.7 nm), Cadmium (wavelength 228.8 nm), Copper (wavelength 324.8 nm), Cobalt (wavelength 240.7 nm), Iron (wavelength 248.3 nm), Manganese (wavelength 279.5 nm), Magnesium (wavelength 285.2 nm), Nickel (wavelength 232.0 nm), Lead (wavelength 283.3 nm) and Zinc (wavelength 213.9 nm) specific hollow cathode lamp was used to analyze the samples. The instrument has a minimum detection limit of 0.06 mg/L for Ca, 0.01 mg/L for Cd, 0.03 mg/L for Cu, 0.05 mg/L for Co, 0.04 mg/L for Fe, 0.02 mg/L for Mn, 0.01 mg/L for Mg, 0.06 mg/L for Ni, 0.20 mg/L for Pb, and 0.01 mg/L for Zn in the flame method. Samples were aspirated through a nebulizer and the absorbance was measured with a blank as reference. A calibration curve was obtained using standard samples. Standard curve concentrations for each metal are shown in (Table 3). The correlation coefficient was found for Ca - 0.998, Cd - 0.998, Cu - 0.999, Co - 0.998, for Fe - 0.996, Mn - 0.998, Mg - 0.999, Pb - 0.999, and Zn - 0.996. The sample had to be diluted many folds to keep the results in the analytical range. Analyzed data for metal concentrations have been shown in (Table 4) with a summary of sampling points.

**Table 3:** Calibration Standard curve concentration range for different metal analysis of seawater.

Standard curve concentration for each metal										
SL. No.	Ca mg/L	Cd mg/L	Cu mg/L	Co mg/L	Fe mg/L	Mn mg/L	Mg mg/L	Ni mg/L	Pb mg/L	Zn mg/L
**1**	1.0	0.2	0.2	0.2	0.5	0.2	1.0	0.2	0.5	0.2
**2**	2.0	0.4	0.4	0.4	1.0	0.4	2.0	0.4	1.0	0.4
**3**	3.0	0.6	0.6	0.6	1.5	0.6	3.0	0.6	2.0	0.6
**4**	4.0	0.8	0.8	0.8	2.0	0.8	4.0	0.8	3.0	0.8
**5**	5.0	1.0	1.0	1.0	3.0	1.0	8.0	1.0	4.0	1.0
**6**	10.0						10.0

**Table 4:** Coordinates of water samples including date, time of sampling, sample depth and tidal condition, metal concentration values from water sample including TOC (Total Organic Carbon). BDL (Below Detection Limit), * = Below Detection Limit.

				Coordinates			Metal concentration										

St. no.	Date	Time (24H)	Sample depth (m)	Latitude (N)	Longitude (E)	Tide	Ca (mg/l)	Cd (mg/l)	Cu (mg/l)	Co (mg/l)	Fe (mg/l)	Mn (mg/l)	Mg (mg/l)	Ni (mg/l)	Pb (mg/l)	Zn (mg/l)	TOC as NPOC (ppm)
1	18/01/16	9:10	2	20.93668	91.570783	High	3.17	3.06	0.04	0.17	0.16	0	*	0.19	0.25	0	1.98
4	20/01/16	14:20	20	20.95002	91.62475	Low	2.68	0.05	0.04	0.21	0.15	0	*	0.2	0.3	0	1.586
7	21/01/16	9:30	10	20.9593	91.661433	High	2.98	0.1	0.07	0.32	0.26	0.1	*	0.33	0.52	0	1.111
8	21/01/16	16:50	20	20.886	91.728567	High	2.94	0.06	0.03	0.21	0.16	0	*	0.22	0.25	0	1.436
10	23/01/16	9:08	5	20.48467	91.008733	High	3.29	0.08	0.05	0.28	0.21	0.1	*	0.29	0.42	0.2	1.605
12	23/01/16	10:31	5	20.51002	91.9628	High	5.66	0.1	0.07	0.35	0.34	0.1	*	0.35	0.53	0.1	2.947
14	23/01/16	11:40	5	20.47147	91.984433	Low	3.45	0.07	0.04	0.25	0.19	0.1	*	0.25	0.29	0	1.89
19	23/01/16	16:25	5	20.63475	91.886883	High	3.17	0.06	0.04	0.21	0.14	0.1	*	0.23	0.31	BDL	1.403
22	24/01/16	9:09	5	20.92175	91.803617	Low	3.62	0.07	0.05	0.25	0.19	0	*	0.11	0.37	0	1.191
26	25/01/16	17:25	5	20.73697	91.87345	High	3.24	0.07	0.04	0.25	0.19	0	*	0.1	0.35	BDL	1.689
28	26/01/16	17:20	5	21.01355	91.51135	High	3.09	0.05	0.03	0.16	0.13	0	*	0.18	0.27	0	1.48
30	26/01/16	21:10	5	20.6106	91.925783	High	4.16	0.09	0.06	0.35	0.3	0.1	*	0.35	0.55	0	1.969
34	28/01/16	14:40	5	20.73723	91.918367	Low	4.28	0.08	0.06	0.3	0.25	0.1	*	0.3	0.47	0.1	2.008
35	28/01/16	16:15	3	20.67965	91.908567	Low	4.03	0.06	0.05	0.26	0.17	0	*	0.26	0.44	0.2	1.793
36	28/01/16	17:30	5	20.69928	91.966617	Low	5.79	0.08	0.06	0.31	0.2	0.1	*	0.31	0.47	0.2	2.134
39	29/01/16	9:40	3	20.89877	91.831717	High	3.22	0.07	0.04	0.24	0.15	0.1	*	0.25	0.35	0.1	1.712
46	30/01/16	10:20	5	20.76478	91.824467	High	3.99	0.08	0.07	0.31	0.26	0.1	*	0.33	0.5	0.1	1.799
47	30/01/16	10:45	5	20.75238	91.774483	High	3.14	0.07	0.05	0.27	0.22	0.1	*	0.27	0.46	0.1	1.926
48	30/01/16	11:25	5	20.69275	91.675517	High	3.45	0.07	0.05	0.25	0.2	0.1	*	0.24	0.42	0.1	2.751
49	30/01/16	11:50	5	20.72958	91.6501	High	2.88	0.07	0.03	0.18	0.12	0	*	0.2	0.3	0	1.852
50	30/01/16	12:05	5	20.7653	91.635767	High	2.62	0.05	0.03	0.16	0.12	0	*	0.17	BDL	0	1.537
51	30/01/16	12:20	5	20.81395	91.616283	High	2.66	0.06	0.03	0.22	0.17	0	*	0.24	0.23	0.1	1.104
52	30/01/16	13:50	5	20.03655	91.550167	Low	3.19	0.05	BDL	0.19	0.13	0	*	0.2	0.29	0.1	1.163
60	1/2/2016	10:00	5	20.43175	92.000317	High	3.75	0.1	0.06	0.37	0.29	0.1	*	0.36	0.49	0.1	1.451
67	1/2/2016	15:55	35	20.5225	91.99015	Low	4.68	0.1	0.06	0.34	0.23	0.1	*	0.33	0.49	0	2.606
68	2/2/2016	11:05	5	20.57198	91.921317	High	3.27	0.08	0.04	0.29	0.23	0.1	*	0.29	0.44	0	5.01
69	2/2/2016	11:45	25	20.5996	91.90735	High	3.79	0.08	0.05	0.33	0.25	0.1	*	0.32	0.49	BDL	1.271
73	3/2/2016	16:05	15	20.97767	91.797	High	3.93	0.1	0.05	0.34	0.25	0.1	*	0.33	0.48	0	1.728
75	10/2/2016	16:43	15	20.62228	91.856433	Low	3.29	0.06	0.04	0.23	0.17	0	*	0.23	0.36	BDL	1.353
76	11/2/2016	10:05	1	20.95367	91.75765	Low	2.44	0.04	BDL	0.22	0.13	0	*	0.15	BDL	0	1.922
Relative error							4.11	57.93	4.86	4.30	5.31	5.51	–	5.17	4.54	18.05	7.405

**Table 5:** Sample station's all relevant data including measurement value of nutrients and chlorophyll in the study area of north – east part of BoB.

				Coordinates					Nutrients				
St. No.	Date	Time (24H)	Sample depth (m)	Latitude (N)	Longitude (E)	Tide	Chl-a mg/m^3^	Phaeopigment	Nitrate (mg/l)	Nitrite (mg/l)	Phosphate (mg/l)	Ammonia (mg/l)	Silica (mg/l)
1	18/01/16	12.10	2	20.93668	91.57078	Low	0	0.416	1.1	2	1.5	3.74	2
2	18/01/16	12.30	10	20.93993	91.57673	Low	0	0.213	2	5.66	0.8	6.09	1
3	20/01/16	13:20	10	20.95002	91.62475	High	0.32	0.096	2.63	2	0.73	5.79	2
4	20/01/16	14:20	20	20.95002	91.62475	High	0	0.208	3	2	0.8	6.51	2
5	20/01/16	18:20	14	20.94772	91.62267	High	0	0.208	4.56	2	0.7	7.26	1
6	21/01/16	9:30	25	20.9593	91.66143	High	0.315	0.536	2.4	2	0.9	7.26	1
7	21/01/16	9:30	10	20.9593	91.66143	High	0.625	0.843	3.5	1	0.9	6.81	2.33
8	21/01/16	16:50	20	20.886	91.72857	High	0.315	0.572	1.7	1	0.6	6.27	5
9	22/01/16	10:57	5	20.9659	91.6321	Low	0.296	0.744	1.6	1	0.6	6.27	5
10	23/01/16	9:08	5	20.48467	91.00873	High	0.296	0.536	1.8	2	0.7	6.99	5
11	23/01/16	10:17	5	20.51787	91.96605	High	0	1.066	2.1	2	0.8	7.88	6
12	23/01/16	10:31	5	20.51002	91.9628	High	0.623	0.252	2	2	0.4	6.54	4
13	23/01/16	11:05	5	20.48157	91.9502	Low	0.311	0.564	2.3	1	0.5	7.96	4
14	23/01/16	11:40	5	20.47147	91.98443	Low	0.279	0.505	2.5	2	0.7	7.05	4
15	23/01/16	12:10	5	20.52958	91.94043	Low	0.315	0.35	2	2	0.6	4.26	4
16	23/01/16	12:46	5	20.55552	91.92237	Low	0.631	0.256	2.4	0	0.3	8.16	3
17	23/01/16	14:02	5	20.60448	91.89055	Low	0.311	0.564	2.6	1	0.3	7.44	4
18	23/01/16	14:50	5	20.62483	91.8521	High	0.631	1.07	4.1	2	0.7	7.38	5
19	23/01/16	16:25	5	20.63475	91.88688	High	0.837	1.229	3.6	2	0.6	5.19	3
20	23/01/16	17:00	5	20.6157	91.9124	High	0.631	0.41	0.3	4	0.3	6.3	1
21	23/01/16	18:10	5	20.56477	91.9394	High	0.315	0.98	3	2	0.6	7.11	3
22	24/01/16	9:09	5	20.92175	91.80362	Low	0	0.208	2.7	2	0.2	7.11	3
23	24/01/16	11:38	5	20.5975	91.9176	Low	0.296	0.296	2.5	0	0.1	7.38	2
24	25/01/16	13:35	5	20.62958	91.93445	Low	0.296	0.296	1.3	1	0.5	7.38	3
25	25/01/16	17:04	5	20.76022	91.85243	High	0.303	0.729	2.6	2	0.5	6.24	2
26	25/01/16	17:25	5	20.73697	91.87345	High	0.631	0.631	2.1	2	0.7	6.3	2
27	25/01/16	18:20	5	20.73093	91.86373	High	0.296	0.12	1	1	0.8	6.36	1
28	26/01/16	17:20	5	21.01355	91.51135	High	0.947	0.947	2	1	0.9	5.79	3
29	26/01/16	17:50	5	21.0098	91.50837	High	0.631	0.478	1.4	2	0.9	6.72	3
30	26/01/16	21:10	5	20.6106	91.92578	High	1.48	0.232	1.6	2	0.7	7.32	1
31	27/01/16	21:45	5	20.6029	91.97707	High	0.947	0.384	1.6	2	0.8	6.24	2
32	27/01/16	22:11	5	20.59057	92.00645	High	3.339	0.287	1.4	2	0.6	6.93	4
33	27/01/16	22:35	5	20.59878	91.9799	High	1.691	1.161	1.4	1	0.7	6.75	2
34	28/01/16	14:40	5	20.73723	91.91837	Low	1.894	0.341	1.6	2	0.5	7.14	2
35	28/01/16	16:15	3	20.67965	91.90857	Low	0.631	0.256	1.9	2	0.7	6.84	2
36	28/01/16	17:30	5	20.69928	91.96662	Low	0	0.221	0.9	2	0.6	6.84	3
37	29/01/16	8:30	3	20.89647	91.83225	High	0.296	0.296	1.2	1	0.5	6.36	3
38	29/01/16	9:15	3	20.9191	91.81715	High	2.525	4.299	1.2	1	0.7	6.09	3
39	29/01/16	9:40	3	20.89877	91.83172	High	0.631	0.631	1.5	1	0.1	7.02	1
40	29/01/16	10:15	3	20.89823	91.87578	High	0.315	0.536	1.2	1	0.1	5.61	2
41	29/01/16	11:30	5	20.90422	91.82905	High	0	0	1.4	2	0.2	6.9	3
42	29/01/16	14:34	5	20.73122	91.8965	Low	0.592	0.592	1.3	1	0.6	6.45	2
43	29/01/16	15:31	5	20.66135	91.92997	Low	0.296	0.12	1.6	1	0.7	7.26	2
44	29/01/16	17:05	5	20.67238	91.94522	Low	0.631	1.296	1.7	1	0.7	7.6	1
45	29/01/16	17:40	5	20.62575	91.96468	Low	0.888	5.672	2.9	5	0.6	4.59	3
46	30/01/16	10:20	5	20.76478	91.82447	High	0.296	1.992	2	1	0.5	4.83	2
47	30/01/16	10:45	5	20.75238	91.77448	High	0.657	2.116	1.1	1	6.2	4.83	4
48	30/01/16	11:25	5	20.69275	91.67552	High	0.315	0.094	2.2	1	0.2	4.77	6
49	30/01/16	11:50	5	20.72958	91.6501	High	0	0	2	1	0.8	4.71	1
50	30/01/16	12:05	5	20.7653	91.63577	High	0	0	1.8	1	0.5	4.44	5
51	30/01/16	12:20	5	20.81395	91.61628	High	0.315	3.9	2.3	1	0.9	3.69	2
52	30/01/16	13:50	5	20.03655	91.55017	Low	0	2.496	1	2	0.6	3.72	3
53	30/01/16	14:30	5	21.02788	91.48562	Low	0.631	5.955	2.8	1	0.7	0.88	1
54	30/01/16	17:23	5	21.00695	91.5086	Low	0	3.536	2.8	0	0.3	1.39	4
55	30/01/16	18:12	5	21.00705	91.55072	Low	0.631	2.627	1.7	2	0.8	0.17	6
56	31/01/16	8:47	3	20.73862	91.84862	High	1.262	3.397	5.8	2	1.1	4.62	2
57	31/01/16	10:05	5	20.67553	91.88542	High	0.338	2.039	4.4	1	0.6	4.98	1
58	31/01/16	10:30	5	20.6563	91.898	High	0.315	1.681	3.2	1	0.5	5.28	1
59	31/01/16	17:09	5	20.48073	92.01902	Low	0.888	1.928	7.3	1	2.1	5.7	2
60	1/2/2016	10:00	5	20.43175	92.00032	High	0.557	0.166	6.2	1	0.6	5.61	1
61	1/2/2016	10:40	5	20.46288	91.99742	High	0.631	0.256	6.5	1	0.5	6.21	2
62	1/2/2016	11:20	5	20.49393	91.97562	High	0.315	0.98	8.6	2	3.5	6.51	1
63	1/2/2016	11:50	5	20.5076	91.95895	High	0.315	0.98	8.2	2	0.5	6	1
64	1/2/2016	13:05	5	20.55838	91.92785	High	0.278	0.669	9.7	1	3.3	6.84	1
65	1/2/2016	13:30	5	20.57493	91.91785	High	0.315	0.094	7.1	1	1.4	6.9	2
66	1/2/2016	15:08	3	20.53082	91.95253	Low	0.296	0.328	1.8	2	0.5	9.88	1
67	1/2/2016	15:55	35	20.5225	91.99015	Low	0.631	0.034	2	1	0.6	6.72	2
68	2/2/2016	11:05	5	20.57198	91.92132	High	0.947	0.282	2.3	0	1.2	6.3	3
69	2/2/2016	11:45	25	20.5996	91.90735	High	0.947	0.167	2	1	0.4	9.2	0
70	2/2/2016	14:30	15	20.57693	91.98743	High	0.296	0.328	2.9	0	0.5	6.39	1
71	2/2/2016	17:45	15	20.5964	91.93987	Low	0.676	0.201	2	1	0.3	7.44	0
72	3/2/2016	9:30	5	20.67477	91.9513	High	0.888	0.056	4.7	0	3	6.24	0
73	3/2/2016	16:05	15	20.97767	91.797	High	0	0	2.9	2	0.2	6.51	0
74	10/2/2016	11:50	15	20.63593	91.9238	Low	0.315	0.128	4.2	0	2.2	6.81	1
75	10/2/2016	16:43	15	20.62228	91.85643	Low	0.947	0.162	4.7	1	0.3	6.3	0
76	11/2/2016	10:05	1	20.95367	91.75765	Low	1.184	0.976	4.3	0	2	6.09	2
Relative error							11.69	15.53	7.82	7.61	12.15	2.94	7.23

#### 2.2.5 Chlorophyll and nutrient

The method used for measurement of chlorophyll-a and phaeopigment was Double extraction hot and cold treatment spectrophotometric method using 90% ethanol as a solvent and 2 M of HCl [12]. This method was picked out due to the double extraction of pigment. Besides, hot and cold treatment stimulate better extraction so that, little or no pigment left over the filter paper. A Pyrex made dispensing bottle was filled with 90% ethyl alcohol keeping some space below the neck of the bottle. The bottle was then positioned in a water bath at a temperature of 75 °C (strict). Filtration of the water sample was done with the help of a vacuum filtration device using circle shaped Whatman GF/F filter paper (47 mm diameter, 45µ pore size) on the flat circular surface of the filter holder. Care was taken so that the filter paper fits well with the rim of the sample holding cup keeping no gap. The water sample was gently blended by reversing and up-righting the sample bottle 3 times. Immediately measured 500 ml of the mixed sample water and was gently poured inside the sample holding cup (volume taken of sample water depends on the density of the phytoplankton population present in the sample and should be judged by eye observation). Then filtration was done connecting the vacuum suction pipe with the port of the filter holder. After finishing the filtration, using a Millipore pincer the filter paper was rolled and placed inside the bottom of a test tube vertically. For an acceptable mean value of chlorophyll-a concentration, it was replicated 3 times. Placed the 3 test tubes in a still made heavy test tube rack. Then 4 ml hot ethanol (kept in the water bath before the filtration) was added to each test tube containing rolled filter paper and was placed on the rack in a water bath for 3 min. After the period was over the rack was placed under the flow of tap water to cool down carefully (so that no tap water enters the test tubes). Another 3 sets of fresh test tubes were taken to transfer the liquid those keeping the filter papers intact in the first set of test tubes. Further, the process was repeated of hot and cold treatment by adding a 2nd volume of hot ethanol in the test tube containing the filter paper. After cooling in the tap water with the help of a bent needle the rolled filter paper was brought out near the mouth of the test tube and using the pincer squeezed out the absorbed pigment sucked by the filter itself. Then added the liquid to the first set of extracted samples and added some 90% hot ethanol to adjust volume up to 10 ml. This 10 ml of the extracted sample was filtered again with the help of a syringe and 25 mm circle of Whatman GF/F filter paper and it was transferred into another set of 3 test tubes. DR6000™ UV VIS Spectrophotometric reading was taken using a cuvette having a path length of 2.5 cm with the filtered 10 ml sample. Reading was taken first at 665 and 750 nm OD and then added 33 µL of 2 M HCl with the help of a micropipette and was gently mixed. Waited for 1 min and again reading was taken at a wavelength of 665 nm and 750 nm OD. All the OD measurements were taken against a blank using 90% ethanol. Determination of biological and physiochemical parameters including Ammonia (NH_3_^−^*N*), Nitrate (NO_3_^−^*N*), Nitrite (NO_2_^−^), Phosphate (PO_4_^3−^), Silica (SiO_2_), were performed using standardized methods of seawater analysis [13]. Finally, the remaining nutrients were measured, adopting the photometric methods (HACH, USA).

#### 2.2.6 Calculation: chlorophyll-a

Eb = (Eb^665^ – Eb^750^), Ea = (Ea^665^ – Ea^750^), Eb/Ea value must be 1.6 or very closer to this for a more accurate concentration of chlorophyll-a. Chla(μg/l)=29.6(Eb−Ea)×v/V×l,

Phaeopigment(μg/l)=[(20.8Ea×v/V×l)−chla], Where, Eb = OD before adding 2 M HCl, Ea = OD after adding 2 M HCl, *v* = Extracted volume of the pigment in ml, *V* = Filtered volume of sample water in liter, *l* = Path length of the cuvette used to measure OD in cm. Three replicates were taken for each sample water, as *n* = 3, so the mean value of it was the Chl-a and phaeopigment concentration of that sample water.

## Declaration of Competing Interest

The authors declare that they have no known competing financial interests or personal relationships that could have appeared to influence the work reported in this paper.

## Appendix Supplementary materials

Image, application 1

Image, application 2

## References

[1] Bhatla R., Mohanty U.C., Raju P.V.S., Madan O.P. (2004). A study on dynamic and thermodynamic aspects of breaks in the summer monsoon over India. Int. J. Climatol..

[2] Masud-Ul-Alam M., Khan A.I., Sunny S.K., Rahman A., Rahman M.S., Mahmud B., Shaheen A.R. (2020). An exclusive in-situ dataset on physicochemical parameters in the gappy northern Bay of Bengal. Data Br..

[3] Baliarsingh S.K., Lotliker A.A., Sahu K.C., Sinivasa Kumar T. (2015). Spatio-Temporal Distribution of Chlorophyll-a in Relation to Physico-Chemical Parameters in Coastal Waters of the Northwestern Bay of Bengal. Environ Monit Assess.

[4] Alheit J. (2009). Consequences of Regime Shifts for Marine Food Webs. International Journal of Earth Sciences.

[5] Sournia A. (1969). Cycle Annuel Du Phytoplancton Et De La pro-Duction Prirnaue Dans Les Mers Tropicales. Mar. Biol..

[6] Cloern J.E. (2001). Our Evolving Conceptual Model of the Coastal Eutrophication Problem. Mar. Ecol. Prog. Ser..

[7] Turner E., Rabalais N.N., Justic D. (2003). Global Patterns of Dissolved N, P and Si in Large Rivers. Biogeochemistry.

[8] Hebbeln D., Paul A. (2009). Marine Biogeochemical Cycles and Ecosystems and Their Interactions with Climate. Int. J. Earth Sci.

[9] Hossain K.B., Ahmed M.K., Hasan A.A., Nahian S.A., Loodh Rupak, Rani S., Chowdhury K.M.A. (2018). Thermal Inversion, Mixed Layer Depth (MLD) and Barrier Layer Thickness (BLT) Variability and Associated Fluorescence Pattern during Winter in the Northern Bay of Bengal. DEW-DROP.

[10] Chowdhury S.R.A., Zafar M. (2018). Physico–Chemical Parameters of Water and Sediment during Spring Period in Parts off Bay of Bengal, Bangladesh. MOJ Ecol. Environ. Sci..

[11] Sarma V.V.S.S., Rao D.N., Rajula G.R., Dalabehera H.B., Yadav K. (2019). Organic Nutrients Support High Primary Production in the Bay of Bengal. Geophys Res Lett.

[12] Marker A., Nusch E., Rai H., Riemann B. (1980). The Measurement of Photosynthetic Pigments in Freshwaters and Standardization of Methods: conclusions and Recommendations. Arch Hydrobiol Beih.

[13] Grasshoff K., Kremling K., Ehrhardt M. (1999). Methods of Seawater Analysis.

