# Supplementary material for: In-situ datasets of important physical and bio-chemical parameters in the continental shelf of the northern Bay of Bengal
Source: Data Brief. 2021 Mar 15;35:106947. doi: 10.1016/j.dib.2021.106947 (PMC8027273; doi:10.1016/j.dib.2021.106947)
Supplement: Supplementary file 1 [file mmc1.docx]

**CRediT statements:**

**Most Israt Jahan Mili:** Conceptualization; Data curation; Formal analysis; Resources; Methodology; Writing - Original Draft; Visualization; Writing - review & editing.

**Md Kawser Ahmed:** Investigation; Supervision; Project administration; Funding acquisition.

**Md Masud-Ul-Alam:** Writing - review & editing; Visualization.

**Md Hasnain:** Data collection.

**Md. Ashif Imam Khan:** Visualization.

**Rupak Loodh:** Sample collection during cruises.

**Abdullah-Al-Hasan:** Sample collection during cruises.

[**Kazi Belayet Hossain**](https://www.facebook.com/munna.hossain)**:** CTD data processing.

**Sultan Al Nahian:** Sample collection during cruises.
